# Involvement of the p62/NRF2 signal transduction pathway on erythrophagocytosis

**DOI:** 10.1038/s41598-017-05687-1

**Published:** 2017-07-19

**Authors:** Inês B. Santarino, Michelle S. Viegas, Neuza S. Domingues, Ana M. Ribeiro, Miguel P. Soares, Otília V. Vieira

**Affiliations:** 1 CEDOC, NOVA Medical School|Faculdade de Ciências Médicas, Universidade NOVA de Lisboa, 1169-056 Lisboa, Portugal; 2 CNC - Center for Neuroscience and Cell Biology, University of Coimbra, Largo Marquês de Pombal, 3004-517 Coimbra, Portugal; 3 Instituto Gulbenkian de Ciência, Oeiras, Portugal, Rua da Quinta Grande, 6, 2780-156 Oeiras, Portugal

## Abstract

Erythrophagocytosis, the phagocytic removal of damaged red blood cells (RBC), and subsequent phagolysosome biogenesis are important processes in iron/heme metabolism and homeostasis. Phagolysosome biogenesis implies the interaction of nascent phagosomes with endocytic compartments and also autophagy effectors. Here, we report that besides recruitment of microtubule-associated protein-1-light chain 3 (LC3), additional autophagy machinery such as sequestosome 1 (p62) is also acquired by single-membrane phagosomes at very early stages of the phagocytic process and that its acquisition is very important to the outcome of the process. In bone marrow-derived macrophages (BMDM) silenced for p62, RBC degradation is inhibited. P62, is also required for nuclear translocation and activation of the transcription factor Nuclear factor E2-related Factor 2 (NRF2) during erythrophagocytosis. Deletion of the *Nrf2* allele reduces p62 expression and compromises RBC degradation. In conclusion, we reveal that erythrophagocytosis relies on an interplay between p62 and NRF2, potentially acting as protective mechanism to maintain reactive oxygen species at basal levels and preserve macrophage homeostasis.

## Introduction

Removal of damaged/aged red blood cells (RBC) from the circulation occurs through erythrophagocytosis, by tissue-resident macrophages in the spleen, liver and bone marrow^1–4^. Rapid removal of damaged RBC is important for maintenance of iron/heme homeostasis, as the majority of iron required to sustain erythropoiesis is derived from senescent RBC, and defects in erythrophagocytosis can lead to anemia and iron overload^4^.

Previous work identified receptor-ligand interactions and signaling pathways engaged during erythrophagocytosis. Namely, macrophages recognize damaged RBC by a range of senescence markers such as phosphatidylserine (PS), decreased levels of sialic acid, CD47 and binding of autologous immunoglobulins and opsonins^5^. Furthermore, some receptors involved in RBC clearance have also been established. Several *in vitro* studies have shown that PS recognition on the cell surface by stabilin-2 is important for RBC clearance, while others suggested that clearance of aged RBC by macrophages is likely dependent on scavenger receptors rather than specific PS receptors^2, 6, 7^. It is likely that under physiological conditions the engulfment of RBC involves a myriad of receptors including the Fc- and complement- receptors.

Upon RBC recognition, macrophage actin cytoskeleton and cell surface remodeling takes place allowing for the formation of a specialized phagosome known as the erythrophagosome. Following scission from the plasma membrane, phagosomes undergo a maturation process involving a programmed change of their membrane and luminal composition resulting from a highly coordinated series of sequential membrane fusion and fission events with components of the endocytic pathway. Fusion with early-endosomes followed by interactions with late-endosomes and lysosomes culminates in the conversion of the phagosome into a lysosome-like organelle - the phagolysosome. It is within this organelle that RBC undergo degradation allowing for the reutilization of their components^4, 8–10^.

Beyond the involvement of vesicular traffic machinery, some components of the autophagy machinery are also involved in phagolysosome biogenesis, including the microtubule-associated protein 1 light chain 3 (LC3), an autophagy effector recruited to single-membrane phagosomes in a process termed LC3-Associated Phagocytosis (LAP). There is strong evidence to suggest that LAP facilitates rapid phagosome maturation while contributing to the degradation of engulfed phagocytic particles and modulation of immune responses^11–13^. In contrast to canonical autophagy, defined by the formation of a double-membrane autophagosome, LAP is associated with the recruitment of LC3 to single-membrane phagosomes carrying different types of cargo in an Atg5-, Atg7- and Beclin1-dependent manner, independently of the mammalian target of Rapamycin (mTor)-regulated ULK-ATG13-FIP200 complex^11, 14^. Rubicon, an adaptor protein, was also identified as being required for LAP but not for autophagy^12^. NADPH oxidase-2 (NOX2) has also been identified as having a LAP-specific role^12, 15^. It should be noted that this brief description of phagosomal maturation is a gross oversimplification of a highly complex and precisely choreographed process.

Although several studies have focused on intracellular mechanisms of heme trafficking during hemophagocytosis^4, 16^, few have addressed the molecular mechanisms underlying maturation and degradation of phagosomes containing RBC. We have recently shown that phagosomes containing RBC cells mature slower than phagosomes containing IgG-opsonized particles^17^, in keeping with the notion that maturation of the phagosome in macrophages depends on the nature of the ingested cargo^18^.

The present study was designed to identify the molecular machinery involved in maturation of phagosomes containing RBC. Of note, while erythrophagocytosis takes place mainly in erythrophagocytic macrophages such as bone marrow-derived macrophages (BMDM) it can also occur in non-professional phagocytes such as hepatic sinusoidal endothelial cells and vascular smooth muscle cells^2, 19–21^. The process has some similarities with efferocytosis that occurs in pathological states like atherosclerosis and in which smooth muscle cells act as non-professional phagocytes in the arterial wall. In previous work we generated a smooth-muscle cell line that stably expressed Fcγ-RIIA receptors and described its use in studies of erythrophagocytosis^17^. Here we report mechanistic details of erythrophagocytosis by this non-professional phagocytic cell line as well as by primary BMDM. We show that beyond LC3, proteins associated with selective autophagy such as p62/SQSTM1 (Sequestosome 1), NBR1 (Neighbor of Braca 1 gene) and NDP52 (Nuclear dot protein 52)^22–24^ are recruited to phagosomal membranes. The most striking phenotype was observed for p62 that associates preferentially with phagosomes containing RBC rather than to phagosomes containing IgG-opsonized particles. Moreover, we demonstrate that p62 is critical for RBC degradation. We also show that erythrophagocytosis triggers the nuclear accumulation of the transcription factor Nuclear factor E2-related factor 2 (NRF2) with subsequent up-regulation of *p62* expression s. In addition, NRF2 affects RBC degradation and p62 levels suggesting a link between these two molecular players in erythrophagocytosis.

## Results

### The type of phagocytic particle determines the association of p62 with phagosomal membranes

**Figure 1:**
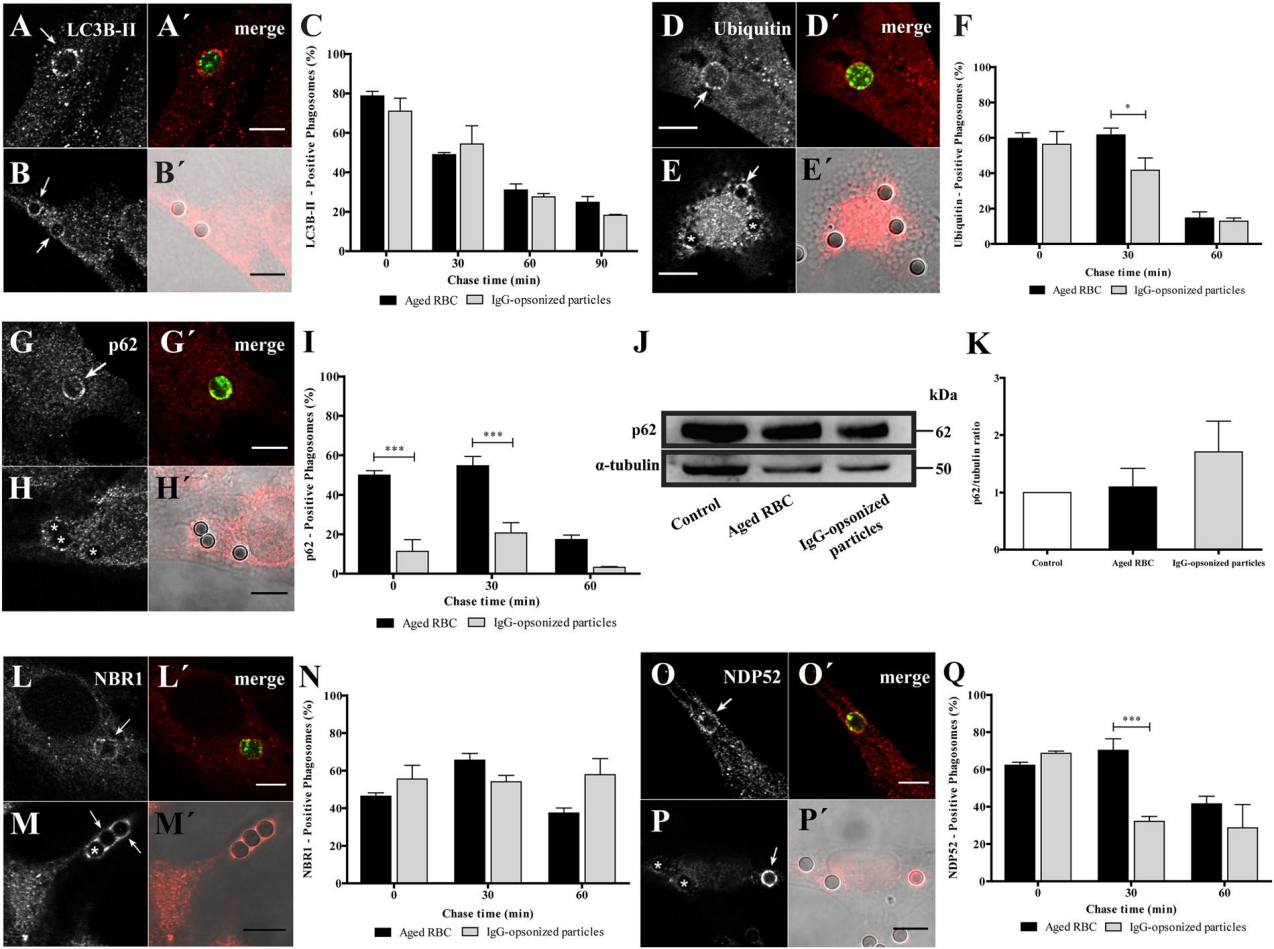
Acquisition of LC3B-II and autophagy adaptor proteins by phagosomes in non-professional phagocytes. Phagocytes were challenged with RBC (**A**,A′,**D**,D′,**G**,G′,**L**,L′ **O** and O′) or with IgG-opsonized particles (**B**,B′,**E**,E′,**H**,H′,**M**,M′,**P** and P′) and then immunostained for the endogenous LC3B, ubiquitin, p62, NBR1 and NDP52 as indicated in the different figure panels. (**A**,**B**,**D**,**E**,**G**,**H**,**L**,**M**,**O** and **P**) are immunofluorescence images. (A′,B′,D′,E′,G′,H′,L′,M′,O′ and P′) are the composite of the immunofluorescence image with the phagocytic particles visualized in green (RBC stained with CFSE) or by differential interference contrast (DIC, IgG-opsonized particles). In (**A**,B′ and **G**,H′) images were acquired at 0 min chase time. In (**D**,E′, **L**,M′ and **O**,P′) the images were acquired at 30 min chase time. Arrows indicate positive- and asterisks (*) indicate negative-phagosomes for the indicated endogenous protein. Bars, 10 µm. (**C**,**F**,**I**,**N** and **Q**), Graphs showing the percentage of positive phagosomes for LC3B-II, ubiquitin and the different adaptor proteins. Quantifications were performed in non-professional phagocytes exposed to the different phagocytic particles for 30 min and chased for the times indicated in the graph abscissa. The values are means ± SEM of, at least, three independent experiments. At each time point, at least, 50 phagosomes were analyzed. *p < 0.05; ***p < 0.001 comparing differences between phagosomes with RBC and with IgG-opsonized particles. (**J**) p62 levels in cell lysates of non-professional phagocytes exposed for 30 min to RBC or to IgG-opsonized particles. α-tubulin was used as loading control. (**K**) Ratio of p62/tubulin of quantified bands in cells exposed or not (control) to RBC or IgG-opsonized particles. Three independent experiments were performed.

We started by studying LAP in the non-professional phagocytes. Damaged/aged RBC were prepared by incubation in PBS (20% hematocrit) for 4 days at 37 °C. This treatment triggers PS-exposure on the outer leaflet of the RBC membrane, resembling what happens to RBC during storage^25^, or eryptosis – a form of programmed cell death similar to apoptosis in nucleated cells^26^. RBC phagocytosis was compared to phagocytosis of IgG-opsonized particles, the most studied phagocytic model. IgG-opsonized particles are known to be internalized via Fc-receptors. After exposing phagocytes to RBC or IgG-coated latex beads, LC3B association with phagosomal membranes was assessed by immunostaining of the endogenous protein by confocal microscopy. LC3B associated with phagosomes containing both particles, immediately after phagocytosis, as evidenced by LC3B-II rims surrounding RBC and opsonized latex beads (Fig. 1A,B′). Both types of phagosomes showed a rapid and transient LC3B membrane association, with peaks reaching a maximum of about 80% (78.7 ± 2.3%) for RBC and about 7% (71.0 ± 6.5%) for opsonized beads, at 0 min chase. These results are in keeping with those reported by other groups showing that LC3B-II can be detected on phagosomes shortly after they are formed while LC3B-II-decorated autophagosomes can take hours to form^11, 27^. LC3B-II gradually dissociated from both types of phagosomes, probably due to recycling from the phagosomal membranes (Fig. 1C). Since our phagocytic assays were performed in serum-free medium and canonical autophagy is activated under conditions of starvation^28^, we tested whether nutrient deprivation was responsible for LC3B-II association with phagosomes containing RBC. As shown in Suppl. Figure 1, no differences in the LC3B-II-phagosomal association pattern were observed when phagocytic cells were kept in the presence or absence of serum, suggesting that LAP machinery is independent of the nutritional status of the phagocytes, as previously described^11, 13^.

Beyond LC3B, phagosomal processing and autophagy share other players and mechanisms^12, 29–32^. Therefore, we monitored phagosomal protein ubiquitination in phagosomes containing both types of phagocytic particles. Phagosomes containing either RBC or IgG-coated beads were associated with poly- and/or mono-ubiquitinated membrane proteins (Fig. 1D–E′). Namely, non-professional phagocytes presented, shortly after ingestion, a large fraction (around 60% for both particles) of phagosomes positive for ubiquitinated components with some differences in the kinetics of signal loss over the maturation time (Fig. 1F), as assessed by the appearance of this tag in phagosomal membranes.

To acquire further insights into the autophagy-related molecular machinery involved in phagolysosome biogenesis, we looked at the phagosomal acquisition of p62, NBR1 and NDP52. These are receptors/adaptors which share the ability to simultaneously interact with the lipidated form of LC3B, LC3B-II and ubiquitinated substrates^31^. We started by testing intracellular distribution of p62 during both types of phagocytosis, a universal receptor for ubiquitinated cargo under physiological and pathological conditions^7, 23, 33–35^. Pulse-chase experiments revealed that RBC containing phagosomes displayed a different pattern of p62 association, as compared to IgG-opsonized beads (Fig. 1G,H). Namely, they showed similar kinetics for acquisition of p62 and ubiquitinated proteins while phagosomes containing IgG-opsonized particles showed only modest levels of p62 over time (compare Fig. 1I and F). That difference was not due to changes in expression levels of total p62 in phagocytic cells challenged with the two phagocytic particles, as confirmed by western blot (Fig. 1J and K).

Next, we analyzed the association of NBR1 with phagosomal membranes. As illustrated in Fig. 1L,M′, NBR1 was recruited to both types of phagosomes. The time course of NBR1 dissociation from membranes of phagosomes that contained RBC was slightly different from the time course of NBR1 dissociation from phagosomes containing IgG-opsonized particles (Fig. 1N). NBR1 dissociation from phagosomal membranes of IgG-coated beads was not observed even for the longest chase time tested. This may be due to a compensatory mechanism for the absence of p62 on the phagosomal membranes of IgG-coated beads.

Finally, we performed immunostaining for NDP52 to assess its acquisition by both types of phagocytic particles (Fig. 1O,P′). Phagosomes containing IgG-opsonized particles showed a transient NDP52 association with around 69% (68.64 ± 1.19%) of positive-phagosomes at 0 min chase and about 29% (28.69 ± 12.51%) at 60 min chase. Phagosomes containing RBC also showed a transient NDP52 association but this autophagy effector remained associated with these phagosomes for longer periods of time compared with those carrying IgG-opsonized beads (Fig. 1Q).

### p62 and NBR1 are recruited to the phagocytic cups

**Figure 2:**
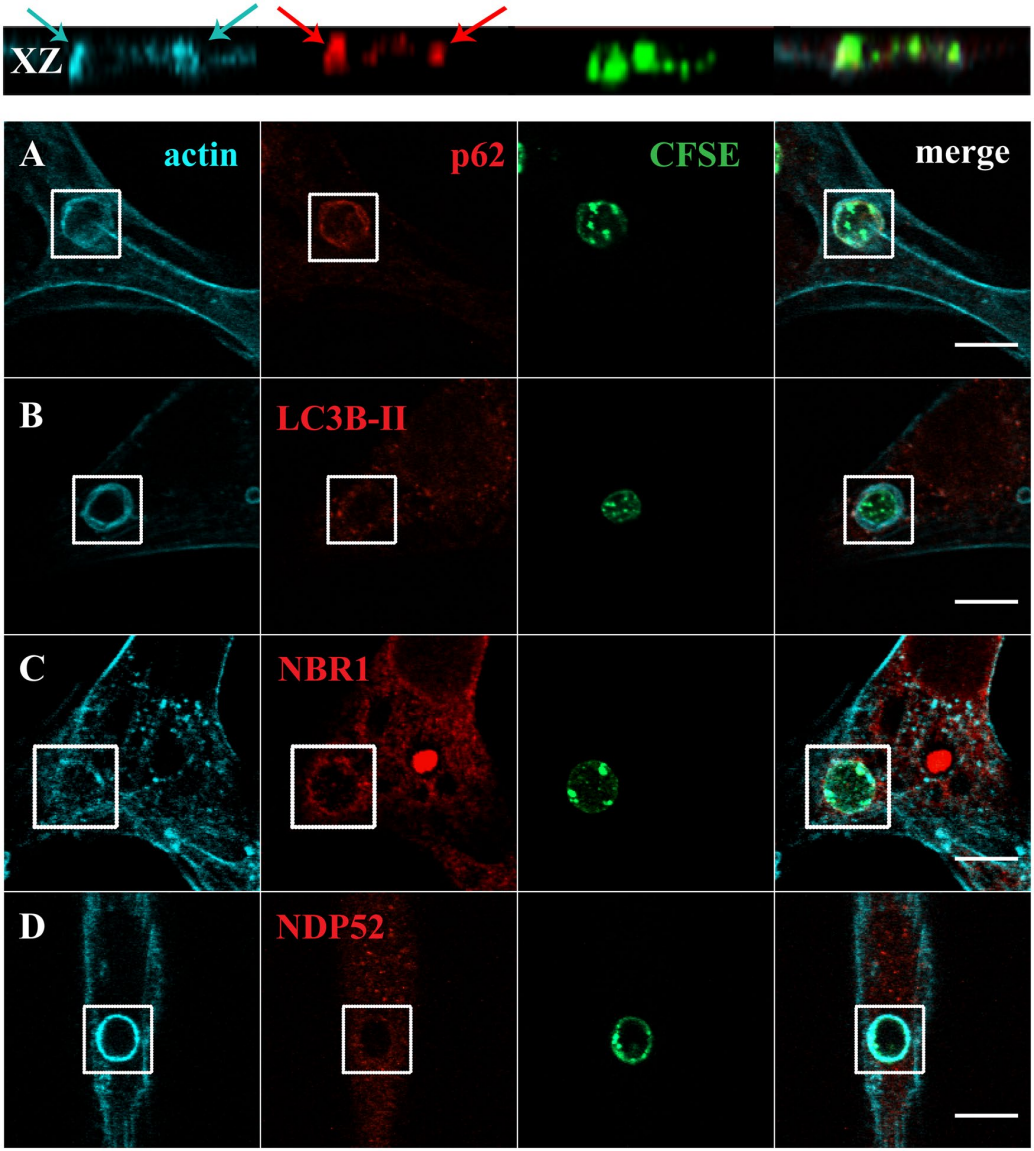
Co**-**localization of LC3B-II and autophagy adaptor proteins with F-actin in phagosomes containing RBC. Non-professional phagocytes were fed with RBC for 30 min, fixed, and stained for F-actin with Phalloidin and for the endogenous LC3B or autophagy adaptors. (**A**–**D**) are representative images, obtained by confocal microscopy, of cells co-stained for F-actin and p62 (**A**), LC3B (**B**), NBR1 (**C**) or NDP52 (**D**). In A, side views (XZ) are merges of ten vertical sections of confocal stacks. Arrows indicate the nascent phagosome positive for F-actin (blue) and p62 (red). The first column represents cells stained for F-actin. The second column represents cells stained for the endogenous p62, LC3B-II, NBR1 or NDP52. The third column shows internalized RBC stained with CFSE. The fourth column represents merged images of F-actin with LC3B-II or autophagy adaptors and internalized RBC. The regions outlined by the boxes are nascent phagosomes. Bars, 10 µm.

**Figure 3:**
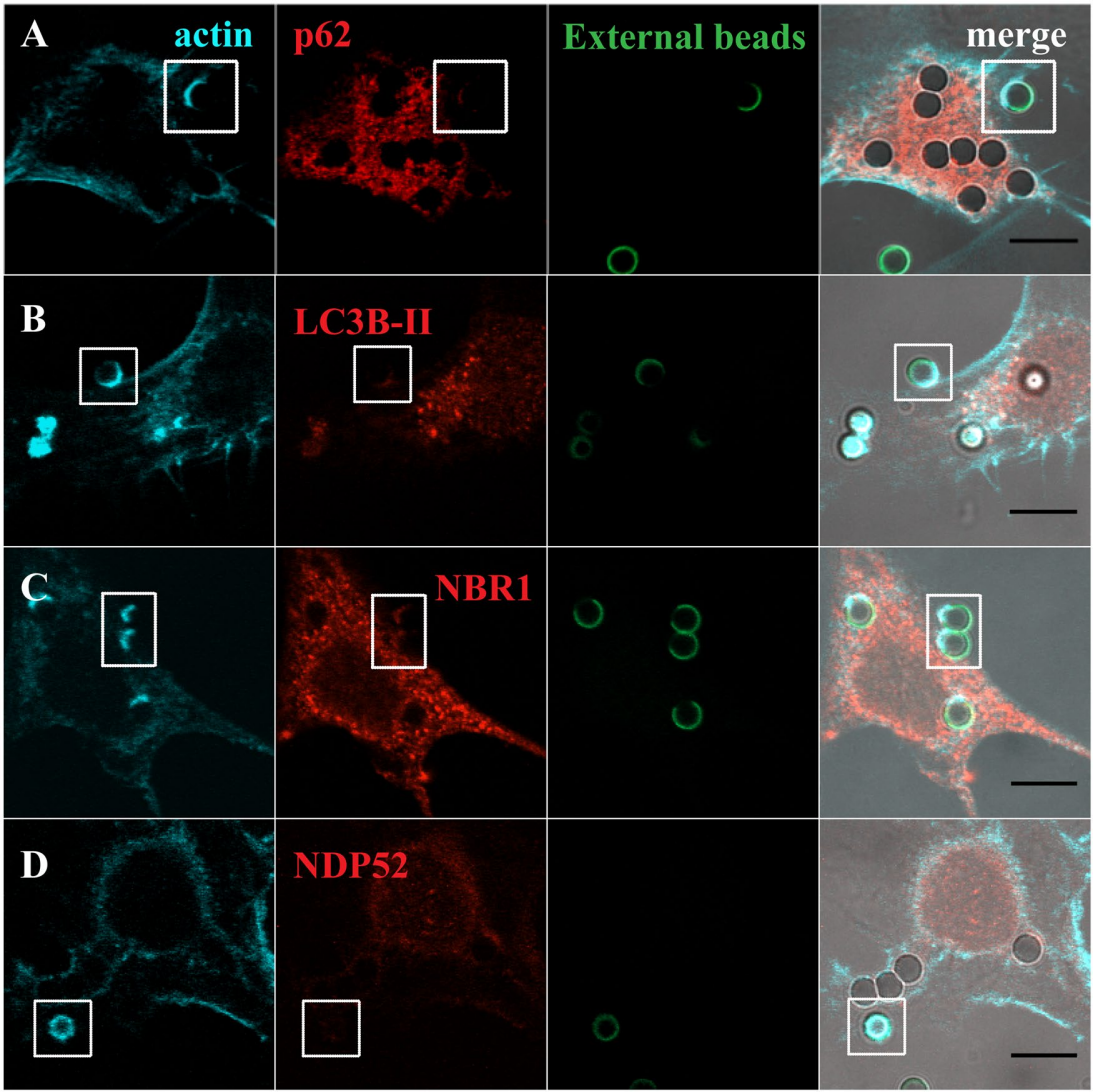
Co-localization of LC3B-II and autophagy effectors in phagocytic cups of IgG-opsonized particles. Non-professional phagocytes were fed with IgG-opsonized beads for 30 min, fixed, and co-stained for F-actin with Phalloidin and for the endogenous LC3B or autophagy adaptors. (**A**–**D**) are representative images of cells co-stained for F-actin and p62 (**A**), LC3B (**B**), NBR1 (**C**) or NDP52 (**D**). The first column represents cells stained for F-actin. The second column represents cells stained for the endogenous p62, LC3B-II, NBR1 or NDP52. The third column shows non-internalized beads stained with an anti-human IgG antibody conjugated with FITC. The fourth column represents merged images of F-actin with LC3B-II or autophagy adaptors and the opsonized latex beads (external and internal beads visualized by DIC). The regions outlined by the boxes are phagocytic cups formed upon the recognition of IgG-opsonized particles by the Fc-receptors. Bars, 10 µm.

Since LAP and the autophagy receptors/adaptors tested in this work were acquired by the phagosomes at very early stages of phagocytosis, we enquired whether they were already present when the phagosomes were positive for F-actin. Phagosome formation is preceded by a dynamic set of events that induce actin cytoskeleton rearrangement in order to support pseudopod extension at sites of particle engulfment. This reorganization leads to a localized cup-shaped protrusion of the plasma membrane, the “phagocytic cup” (Figs 2 and 3). This structure is enriched in actin filaments responsible for generation of the forces that alter the local shape of the cell surface. In the case of phagocytosis of RBC, membrane protrusions are formed upon actin polymerization, with particle sinking followed by the formation of the phagosome, through a process between complement-mediated phagocytosis and micropinocytosis^36^ making the visualization of the phagocytic cups difficult, as can be observed in Fig. 2A (blue arrows in the XZ view point to F-actin). In contrast, in phagocytosis of IgG-opsonized particles, the actin cups are perfectly visualized (Fig. 3).

To assess how early the autophagy receptors/adaptors associate with the phagocyte particles, phagocytes were exposed to RBC and IgG-opsonized beads, fixed and co-stained for F-actin with Phalloidin and p62, LC3B, NBR1 or NDP52. Notably, p62 was found to be present in nascent RBC-containing phagosomes, co-localizing with F-actin as shown in Fig. 2A and absent in phagosomes containing IgG-opsonized particles, Fig. 3A, confirming the selectivity of this adaptor for RBC-containing phagosomes (Fig. 1I). As seen in Figs 2B,D and 3B,D, LC3B-II and NDP52 were not co-localized with F-actin in any of the phagocytic particles incubated with phagocytes suggesting that they were acquired by the phagosomal membranes after actin dissociation and when phagosome maturation starts. NBR1 was present in the phagocytic cups of RBC and IgG-opsonized particles (Figs 2C and 3C). Thus, during the phagocytic process, p62 and NBR1 were acquired earlier that NDP52 and LC3B-II, suggesting that the former proteins could be involved in the recruitment of the latter.

### Ubiquitin is involved in the recruitment of the autophagy adaptors to the phagosomal membranes

**Figure 4:**
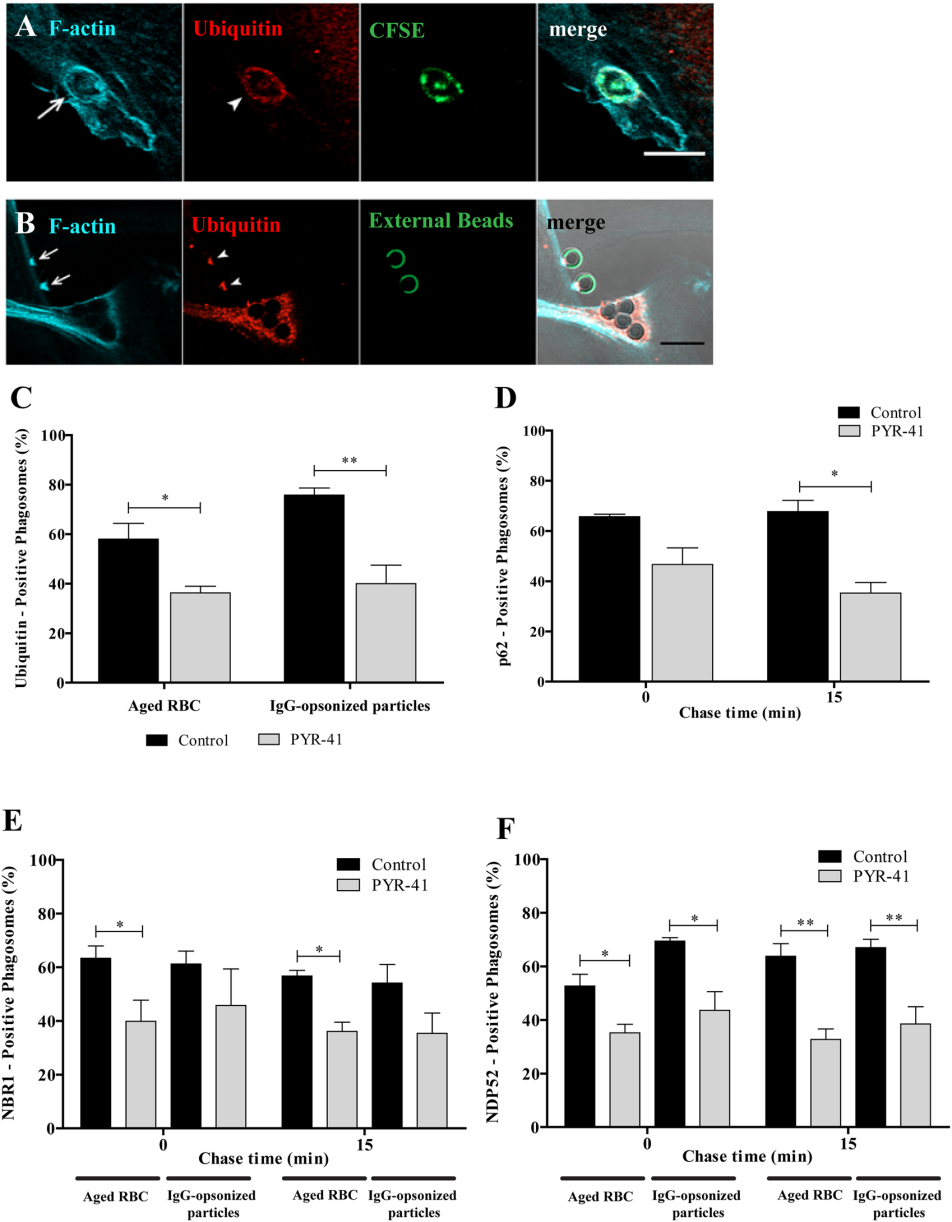
Functional relevance of ubiquitin on the recruitment of autophagy effectors to phagosomes. Non-professional phagocytes were fed with RBC or IgG-opsonized particles for 30 min, fixed, and co-stained for F-actin and ubiquitin. Representative image of a nascent phagosome (**A**) and a phagocytic cup (**B**) positive for actin (first panels) and ubiquitin (second panels). The third panels show internalized RBC labelled with CFSE and non-internalized beads stained with an anti-human IgG antibody conjugated with FITC. The fourth panels are composites of the 1^st^, 2^nd^ and 3^rd^ panels. Arrows and arrowheads indicate actin- and ubiquitin-positive phagosome, respectively. Bars, 10 µm. (**C**) Effect of PYR-41 in the ubiquitination of both RBC- and IgG-opsonized particles-containing phagosomes. Phagocytes were cultured and treated as described in Material and Methods section. (**D**) Quantification of PYR-41 effect on the acquisition of p62 by RBC-containing phagosomes. (**E**,**F**) Quantification of PYR-41 effect on the acquisition of the autophagy adaptor proteins, NBR1 and NDP52, respectively, by RBC- and IgG-opsonized particles-containing phagosomes. The values are means ± SEM of, at least, three independent experiments. At each time point, at least, 50 phagosomes were analyzed. *p < 0.05; **p < 0.01 comparing differences between adaptor-positive phagosomes containing RBC or IgG-opsonized particles in absence and in presence of the inhibitor PYR-41.

Since p62, NBR1 and NDP52 have ubiquitin binding domains and ubiquitination occurs during phagocytosis (Fig. 1D–F), we addressed the role of ubiquitin in the recruitment of these adaptors to phagosomes. First, we determined whether ubiquitin was associating with phagocytic cups. As observed in Fig. 4A and B, ubiquitin associated with phagocytic cups, visualized by F-actin staining, suggesting that phagosomal ubiquitination could be involved in the recruitment of p62, NBR1 and NDP52. An E1 ubiquitin-activating enzyme inhibitor, PYR-41^37^, reduced the percentage of ubiquitin-positive phagosomes containing RBC by 37.0% and by 47.0% for IgG-opsonized particles, when compared with control cells (Fig. 4C). Although inhibition of ubiquitination by PYR-41 of phagosomal membranes was not complete, the effect obtained was sufficient to affect the association of p62 (Fig. 4D), NBR1 (Fig. 4E) and NDP52 (Fig. 4F) with phagosomes containing RBC. The inhibitory effect of PYR-41 in the ubiquitination of phagosomal membranes containing IgG-opsonized particles was only observed for NDP52 recruitment to the phagosomal membranes (Fig. 4E and F). Together, these results suggest a new and differentiating role for ubiquitin in phagocytosis.

**Figure 5:**
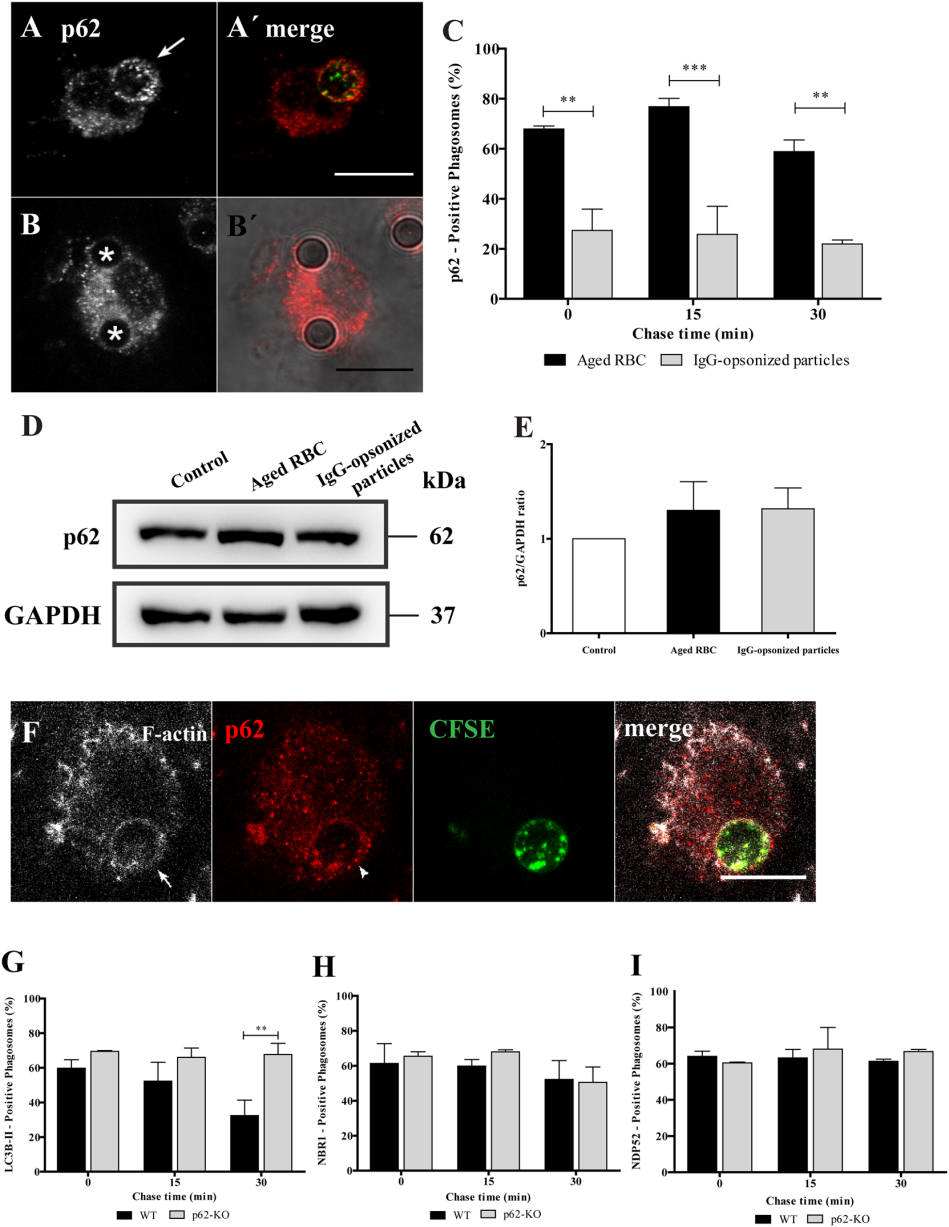
Effect of p62 in the recruitment of LC3B-II, NBR1 and NDP52 to phagosomes containing RBC cells in BMDM. After pulse-chase experiments with RBC or IgG-opsonized particles, WT-BMDM cells were fixed and stained for p62. (**A**) WT-BMDM containing a p62-positive phagosome at 15 min chase time. (A′) Corresponding merged image showing the internalized RBC stained with CFSE. (**B**) WT-BMDM containing p62-negative phagosomes at 15 min chase time. (B′) Corresponding merged image showing the internalized IgG-opsonized particles in DIC. (**C**) Quantification of p62 positive-phagosomes. (**D**) p62 levels in total cell lysates of WT-BMDM exposed for 15 min to RBC or IgG-opsonized particles. GAPDH was used as loading control. (**E**) Ratio of p62/GAPDH of quantified bands in cells exposed or not to RBC, IgG-opsonized particles. Three independent experiments were performed. (**F**) Representative image of RBC-containing phagosome positive for actin (in white) and p62 (in red). Arrow and arrowhead indicate actin- and p62-positive phagosome, respectively. Quantification of LC3B-II- (**G**), NBR1- (**H**) and NDP52- (**I**) positive phagosomes in WT- BMDM (black bars) and p62-KO- BMDM (grey bars). The values are means ± SEM of, at least, three independent experiments. At each time point, at least, 50 phagosomes were analyzed. **p < 0.01; ***p < 0.001 comparing differences between p62-positive phagosomes containing RBC and IgG-opsonized particles or differences between of LC3B-II-positive phagosomes in WT- and p62-KO-BMDM.

The role of p62 in phagocytosis of IgG-opsonized particles and RBC was compared in mouse BMDM. As shown in Fig. 5A–C the results for wild type BMDM exhibit a pattern that is very similar to the one described above for non-professional phagocytes with p62 associated mainly with RBC-containing phagosomes irrespective of the total p62 levels (Fig. 5D and E). Similarly, p62 associates with phagosomal membranes at very early stages of the phagocytic process in BMDM (Fig. 5F). This suggests that the role of p62 is conserved in professional and non-professional phagocytes. Due to the residual levels of p62 detected in phagosomes containing IgG-opsonized particles we explored in further detail the role of p62, focusing only on RBC-containing phagosomes using mouse BMDM silenced for p62 or BMDM from p62-deficient mice (p62^−/−^).

To ensure that RBC internalization and processing was a LAP-dependent process we assessed the recruitment of LC3B to phagosomal membranes in absence of Rubicon, an adaptor protein associated to the LAPosome and required for LAP^12^. As illustrated in Suppl. Figure 2A,B, LC3B-II association with phagosomal membranes was inhibited in absence of Rubicon, when compared to control cells expressing Rubicon. This confirmes that RBC internalization is mediated by LAP.

**Figure 6:**
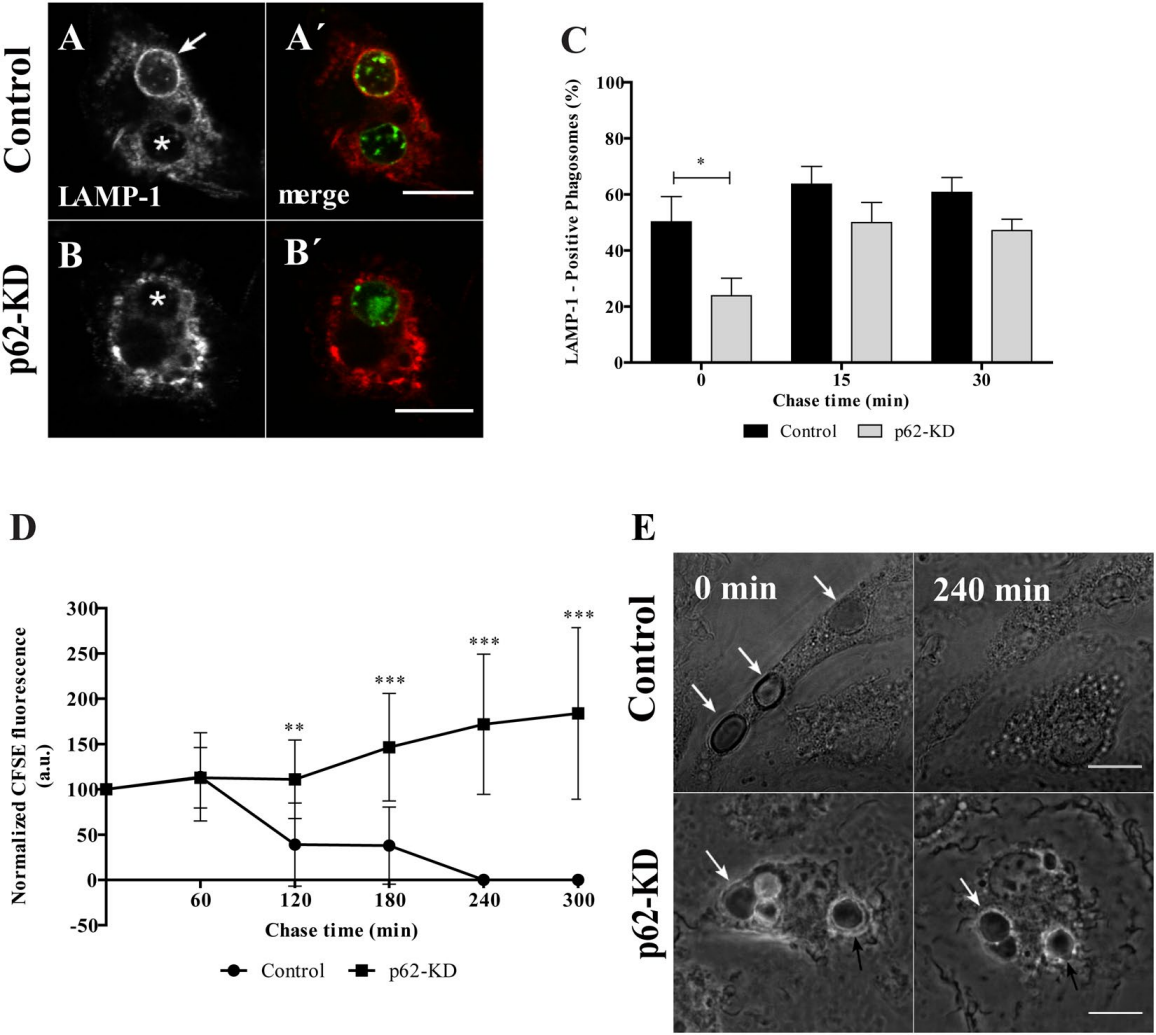
Functional relevance of p62 in RBC degradation. After pulse-chase experiments with RBC, control- and p62-silenced BMDM were fixed and stained for LAMP-1. (**A**) Control-BMDM containing LAMP1-positive and LAMP1-negative phagosomes at 0 min chase time. (**B**) p62-KD-BMDM containing a LAMP-1-negative phagosome at 0 min chase time. (A′,B′) Corresponding merged images showing the internalized RBC stained with CFSE. Arrow indicates a LAMP-1-positive phagosome and asterisks (*) indicate LAMP-1-negative phagosomes. (**C**) Quantification of LAMP-1-positive phagosomes in WT-BMDM (black bars) and p62-KD-BMDM (grey bars). The values are means ± SEM of, at least, three independent experiments. At each time point, at least, 50 phagosomes were analyzed. *p < 0.05, comparing differences between WT- and p62-KD-BMDM. (**D**,**E**) Time-lapse experiments of WT- and p62-KD-BMDM challenged with CFSE labeled-RBC for 15 min (0 min chase time) and followed for 300 min further to assess phagosome degradation. Phagosome degradation (**D**) was measured by the disappearance of fluorescence and the phagosome assessed by DIC (**E**). Bars, 10 µm. The values are means ± SD of 10 different phagosomes. **p < 0.01; ***p < 0.001 comparing differences between WT- BMDM and p62-KD-BMDM. (**E**) DIC images at 0 min and 240 min chase time of WT- BMDM and p62-KD-BMDM. Arrows point to RBC-containing phagosomes.

Because p62 recruitment to the phagosomal membranes preceded that of LC3B-II (Fig. 2A and B), we assessed the requirement of the former in the phagosomal association of the latter. Figure 5G compares LC3B-II dissociation from phagosomal membranes of wild type (*p62* ^+/+^) and *p62* ^−/−^ BMDM and shows that in *p62* ^−/−^ BMDM LC3B-II did not dissociate from these membranes over the periods of time examined, as compared to *p62* ^+/+^ BMDM. As p62 can interact with NBR1 and NDP52^38^, we tested whether recruitment of these autophagy effectors to phagosomes was dependent on p62. As shown in Fig. 5H and I, neither NBR1 nor NDP52 association with phagosomal membranes required p62. Interestingly, the effect of p62 absence in LAP (Fig. 5G) delaying LC3B-II dissociation/degradation from the phagosomal membranes, seemed to have consequences in phagosome maturation and degradation. Phagolysosome biogenesis was assessed by the acquisition of the lysosomal membrane marker LAMP-1 in p62 silenced cells, in which p62 expression was reduced by 70.4 ± 0.08% assessed by qPCR and by Western blot (see Suppl. Fig. 2C,D). As illustrated in Fig. 6A–C, absence of p62 caused only a delay in LAMP-1 acquisition. Indeed, the percentage of LAMP-1-positive phagosomes in p62 silenced cells was only lower when compared to the phagosomes in control cells at 0 min chase time (23.8 ± 6.3% compared with 50.2 ± 9.1%, respectively).

This effect on phagolysosome biogenesis can have an impact in the RBC degradation which in turn can lead to a defective uptake and subsequent oxidative damage. Indeed, the absence of p62 impaired RBC degradation (Fig. 6D and E). Control and p62-silenced cells were challenged with CFSE-labeled RBC for 15 min and then followed by live-cell confocal microscopy for 300 min. In control cells, the internalized RBC underwent efficient degradation after 60 min chase (assessed by the loss of fluorescence and disappearance of RBC by DIC, Fig. 6D and E upper panels, respectively). In contrast, the absence of p62 led to the failure of RBC degradation showed by the persistence of these RBC even after 300 min chase (Fig. 6D and E lower panels). This suggests that p62 is critical for phagosomal maturation and RBC degradation.

### Erythrophagocytosis is associated with p62-dependent NRF2 activation

**Figure 7:**
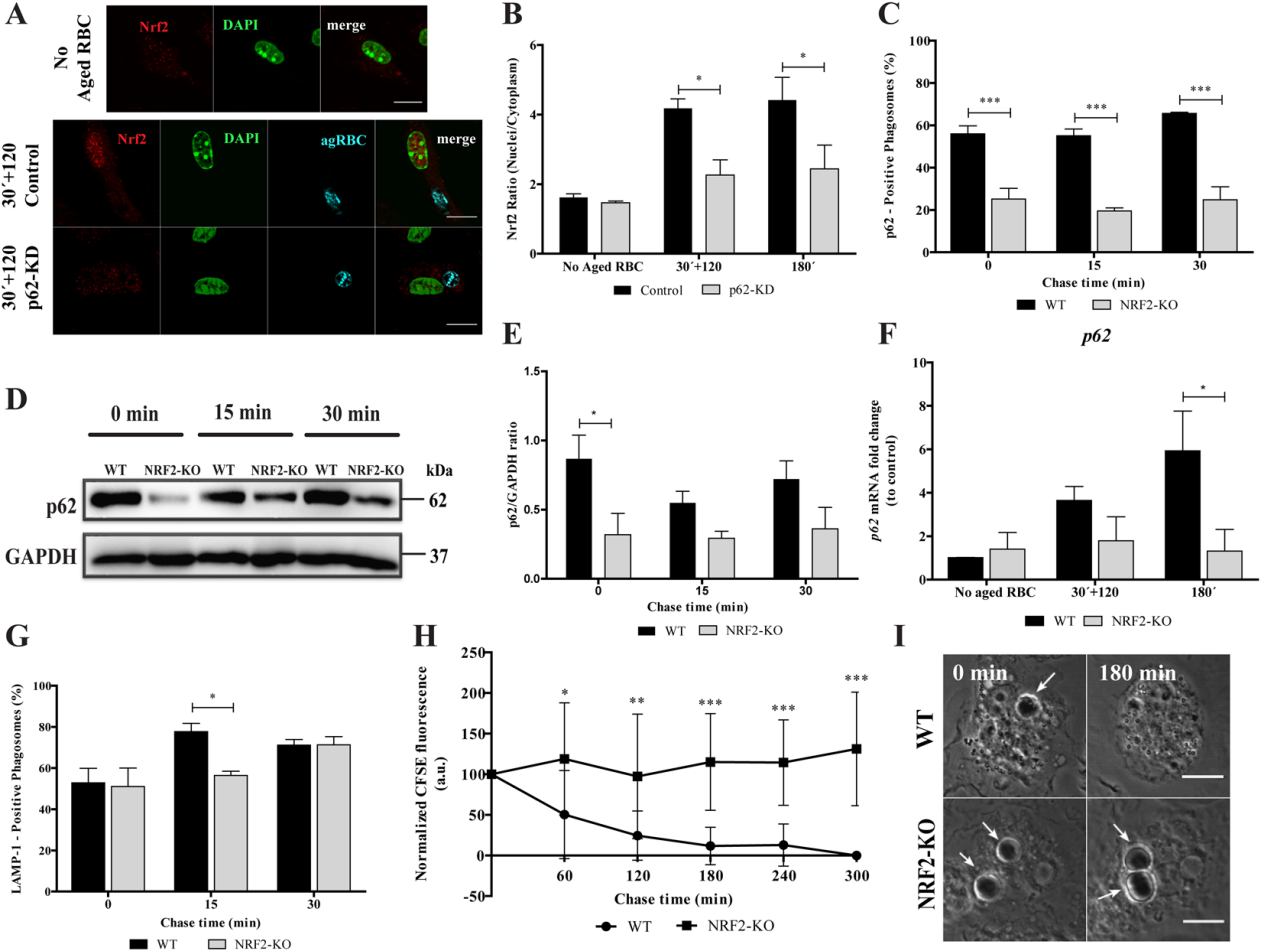
NRF2 is critical for RBC degradation in BMDM. WT- and p62-KD-BMDM were fed with CFSE-stained RBC for 30 min and then chased for 120 min or fed for 180 min. (**A**) Translocation of NRF2 into the nucleus in WT- and in p62-KD-BMDM, assessed by immunostaining, in the absence (first row) or upon incubation with RBC (second and third rows). The second row represents NRF2-nuclear translocation in WT-BMDM. The third row represents NRF2-nuclear translocation in p62-KD-BMDM. NRF2 staining is represented in red, nucleus in green and internalized RBC in cyan. The last panels are merged images. Bars, 10 µm. (**B**) Quantification of NRF2 nuclear translocation expressed as a ratio of the fluorescence intensity between the nucleus and the cytoplasm. *p < 0.05 comparing differences between NRF2 fluorescence in WT-BMDM and p62-KD-BMDM challenged with RBC. (**C**–**F**) WT- and NRF2-KO-BMDM were challenged with RBC for 15 min (0 min chase) and then chased for the indicated times in the figures. (**C**) Quantification of p62-positive phagosomes after fixation and immunostaining for the endogenous protein. (**D**) p62 levels in total cell lysates of WT- and NRF2-KO-BMDM, for short time points. GAPDH was used as loading control. (**E**) Ratio of p62/GAPDH of quantified bands in cells exposed to RBC. Three independent experiments were performed. (**F**) WT- and NRF2-KO-BMDM were challenged with RBC for 30 min and then chased for 120 min or fed for 180 min. The expression of *p62* gene was assessed by RT-qPCR. Data were normalized to the endogenous *Hprt* and *Pgk1* genes. The values are means ± SEM expression levels of three independent experiments, each measured in two technical replicates. *p < 0.05. (**G**) Quantification of LAMP-1-positive phagosomes after fixation and stained for LAMP-1. The values are means ± SEM expression levels of three independent experiments, each measured in two technical replicates. *p < 0.05. (**H**,**I**) Time-lapse experiments of WT- and NRF2-KO-BMDM challenged with CFSE labeled-RBC for 15 min (0 min chase time) and followed for 300 min further to assess phagosome degradation. Phagosome degradation was analyzed as described in the legend of Fig. 6. The values are means ± SD of 10 different phagosomes. *p < 0.05; **p < 0.01; ***p < 0.001 comparing differences between WT- and NRF2-KO-BMDM. (**H**) DIC images at 0 min and 180 min chase time of WT- and NRF2-KO-BMDM. Arrows point to RBC-containing phagosomes.

Under basal conditions, NRF2 is ubiquitinated by Kelch-like ECH-associated protein 1 (KEAP1)-Cul3-E3 ubiquitin ligase complex and targeted to the 26 S proteasome for degradation. Oxidative stress represses KEAP1 binding to the Cul3–Rbx1 complex, allowing newly transcribed NRF2 to undergo nuclear translocation^39–41^. NRF2 can also be activated via a non-canonical mechanism: phosphorylation of Ser351 on the KIR domain (Keap1-interacting region, aa 346–359) of p62 causing p62’s affinity for KEAP1 to significantly increase^42^. Upon nuclear translocation NRF2 heterodimerizes with other basic leucine zipper transcription factors, such as small musculoaponeurotic fibrosarcoma (Maf) proteins and binds to antioxidant response element (ARE) in the promoter of NRF2-regulated genes to induce their transcription expression^43–45^. To acquire more mechanistic insights concerning the role of p62 in erythrophagocytosis we questioned whether erythrophagocytosis was associated with p62-dependent nuclear NRF2 accumulation. As illustrated in Fig. 7A,B, RBC internalization was associated with NRF2 nuclear accumulation, later in the phagocytic process, reaching maximum at 30 min pulse followed by 120 min chase or at 180 min. The fluorescence intensities ratio of NRF2 on nuclei and cytoplasm was roughly twofold in WT-BMDM exposed to RBC compared to unstimulated controls (Fig. 7A and B). This suggests that NRF2 nuclear translocation occurs only after fusion of phagosomes with lysosomes upon RBC degradation (Fig. 6C and D). In absence of p62, NRF2 nuclear translocation was reduced by 50%, when compared with control cells expressing p62 (Fig. 7A and grey columns in B). Next, we assessed the role of NRF2 on phagosomal maturation and RBC degradation, using BMDM from *Nrf2* ^−/−^ mice. Through the entire maturation process the percentage of p62-positive phagosomes in *Nrf2* ^−/−^ BMDM was reduced (Fig. 7C) when compared with *Nrf2* ^+/+^ BMDM. This could be attributed to lower levels of p62 in *Nrf*2^−/−^ BMDM when compared with *Nrf2* ^+/+^ BMDM as shown in the Western Blot (Fig. 7D) and quantified in Fig. 7E. Finally, we assessed whether NRF2 nuclear translocation during erythrophagocytosis is associated with p62 transcription (Fig. 7F). P62 expression increased after NRF2 translocation and absence of NRF2 showed a significant inhibition of p62 expression (5.93 ± 2.23 *versus* 1.31 ± 1.23) at 180 min of erythrophagocytosis. Altogether, these results strongly suggest the existence of a positive feedback between NRF2 and p62 in which p62 is required for NRF2 nuclear translocation during erythrophagocytosis, which induces the levels of P62 expression.

Transport of RBC-containing phagosomes to the lysosomes was only slightly affected in *Nrf2* ^−/−^ vs. *Nrf2* ^+/+^ BMDM (Fig. 7G), while the effect on RBC degradation was very striking (Fig. 7H and I). These two outcomes were similar to the results obtained in p62 silenced BMDM (Fig. 6D and E), suggesting that p62 and NRF2 are involved in the same pathway.

**Figure 8:**
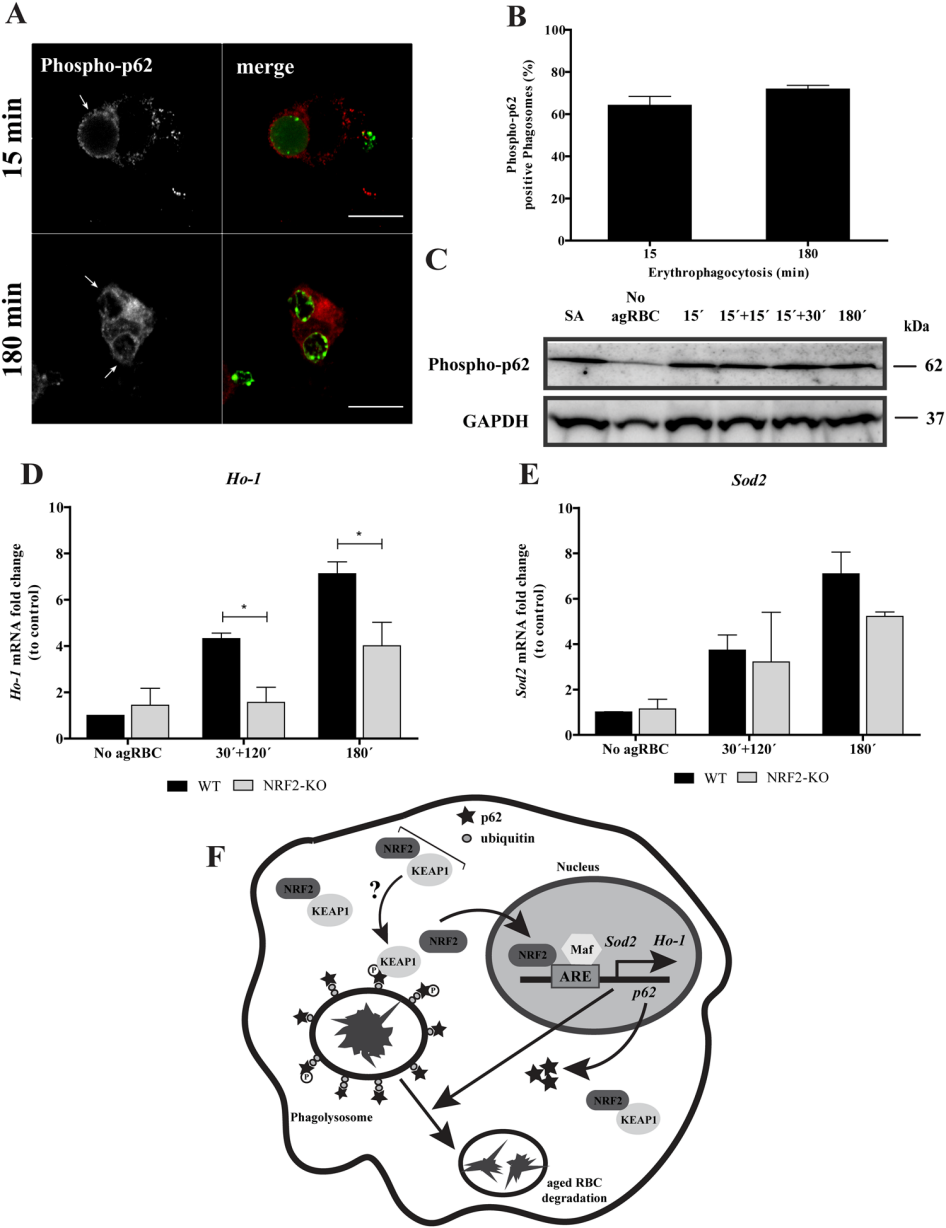
p62 Phosphorylation and NRF2-target genes expression upon erythrophagocytosis. WT-BMDM were fed and chased with CFSE-labeled RBC for the times indicated and imunostained for phospho-p62 (**A**,**B**) Quantification of phosphorylated p62-positive phagosomes. The values are means ± SEM expression levels of 2 independent experiments. (**C**) phosphorylated-p62 levels in cell lysates exposed to RBC. GAPDH was used as loading control. Two independent experiments were performed. As positive control, Sodium arsenite was added for 12 h to a final concentration of 10 µM. In D-E, WT- and NRF2-KO-BMDM were challenged with RBC for 30 min and then chased for 120 min or fed for 180 min. The expression of *HO-1* (**D**), *SOD2* (**E**) genes was assessed by RT-qPCR. Data were normalized to the endogenous *Hprt* and *Pgk1* genes. The values are means ± SEM expression levels of three independent experiments, each measured in two technical replicates. *p < 0.05. (**F**) Working Model: Our model suggests that degradation of RBC by macrophages is dependent on p62/NRF2 signaling pathway. p62 is recruited to RBC-containing phagosomes decorated with ubiquitin shortly after erythrophagocytosis, followed by phosphorylation in the S351 residue and KEAP1 acquisition, by an unknown mechanism. Then, after phagolysosome formation NRF2 is translocated to the nucleus where together with small Maf proteins, binds to ARE in promoter region of *Ho-1*, *p62* and *Sod2* genes, inducing their expression. This molecular machinery promotes aged RBC degradation and controls oxidative stress. NRF2 - nuclear factor erythroid derived 2-like 2; KEAP1 - Kelch-like ECH-associated protein 1; ARE – antioxidant response element; Maf - small masculoaponeurotic fibrosarcoma; *Sod2* - superoxide dismutase 2; *Ho-1* - hemoxigenase 1.

The non-canonical NRF2-signaling pathway activation^42^ is associated with p62 phosphorylation at S351, by mTORC1, decreasing NRF2 binding affinity towards KEAP1 and therefore inducing the expression of NRF2-target genes. As illustarted in the Fig. 8A and quantified in the Fig. 8B, p62 S351 phosphorylation occurred in the phagosomal membranes containing RBC at early time points and had a tendency to increase with time. In BMDM challenged with agRBC an increase in the total p62 S351 phosphorylation was observed when compared to BMDM not exposed to agRBC (Fig. 8C).

Since heme is a pro-oxidant molecule, we tested the involvement of NRF2 in the transcription of anti-oxidant genes, including thioredoxin-1, ferritin, glutathione S-transferase, peroxidoxin-1, heme oxygenase 1 (*Hmox1*), catalase, superoxide dismutase (*SOD*) 1 and 2. Among these, only the expression of *Hmox1* and *Sod2* were increased after NRF2 translocation in BMDM challenged with RBC (Fig. 8D and E, black bars). As observed in Fig. 8D, HO-1 expression was reduced in *Nrf2* ^−/−^ *vs. Nrf2* ^−/−^ BMDM, after erythrophagocytosis. Although, no statistical significances were observed for *Sod2* gene expression (Fig. 8E), it was possible to see a slight difference at 180 min pulse time in *Nrf2* ^−/−^ BMDM when compared with Nrf2^+/+^ BMDM. Thus, the involvement of NRF2 on *Hmox1* and *Sod2* expression can be attributed to the intracellular release of potentially pro-oxidant labile heme, which occurs after RBC degradation within the lysosomes. Accordingly, we conclude that NRF2 is both part of the machinery required for RBC degradation as well as for the anti-oxidative response.

## Discussion

While erythrophagocytosis is critical to the regulation of iron/heme metabolism and maintenance of homeostasis, our understanding of the molecular processes underlying the maturation of phagosomes containing RBC and their subsequent degradation by hemophagocytic macrophages is quite rudimentary. In this work we provide some new insights into the biogenesis of phagolysomes containing RBC, their maturation, and the ordered degradation of RBC in both non-professional and professional phagocytes. We show that the process is complex and involves a convergence of endocytic and autophagic processes. When RBC are phagocytosed, p62 and NRF2 are critical for phagolysosome biogenesis and degradation. Our findings also show that beyond the involvement of LC3B-II, other components of the selective autophagy machinery such as NBR1, NDP52 and p62 are also recruited to the single membrane phagosomes.

Among the selective autophagy machinery tested, the most interesting outcome was observed for p62. This protein is recruited mostly to phagosomes carrying RBC but very weakly to phagosomes containing IgG-opsonized particles, suggesting not only that it has a negligible role in Fc-mediated phagocytosis but also the existence of different types of LAP. The fact that these results were observed in both non-professional and professional phagocytes suggests a conserved role of p62 in RBC-containing phagosomes maturation and degradation. p62 associates with phagosomal membranes at very early stages of the RBC-phagocytic process, co-localizing with F-actin. We attempted to understand what signals the association of p62 with the phagocytic cups. Among other domains, p62 has a LC3-interacting motif (WXXL/I) called the LC3-recognition sequence (LRS) or the LC3-interacting region (LIR) as well as ubiquitin binding domains^22, 46, 47^. Our results indicate that p62 is not recruited to phagosomal membranes via interaction with LC3B-II since this protein associates with the phagosomal membranes after p62 and when F-actin is no longer detected on phagosomes. Using a pharmacological approach, we found that the recruitment of p62 might rely, at least in part, via the interaction of its ubiquitin-binding domain with ubiquitinated components of the phagosomal membranes.

The absence of p62 affects phagolysosome biogenesis with striking effects on RBC degradation. Though p62 has been shown to mediate intracellular xenophagic degradation of bacteria that undergo ubiquitination in response to their escape from phagosomes and subsequent formation of a double membrane organelle^33, 35, 48^, to our knowledge this is the first time that the requirement of p62 for phagocytic cargo degradation within a single membrane organelle is reported. Interestingly, when RBC within the phagolysosome start to be degraded, NRF2 translocates to the nucleus. The translocation of this transcription factor is p62-dependent and in its absence, RBC degradation is blocked suggesting that p62 and NRF2 act together to degrade these phagocytic particles. We enquired how and why p62 and NRF2 interact upon erythrophagocytosis. In xenophagy, p62 is translocated to autophagosomes containing invasive *Salmonella* leading to Ser351 phosphorylation in the KIR motif, enhancing the interaction between p62-KEAP1 and consequently NRF2 translocation^49^. Furthermore, under amino acid rich conditions the mTORC1 has been identified as one of the kinases responsible for the phosphorylation of p62^42^. Interestingly, NRF2-target genes are induced at the same time that autophagosomes entrap the microbes^49^. Our data, however, reveal a different sequential dynamic of p62 and NRF2 in response to RBC engulfment. In erythrophagocytosis, association of p62 with phagosomal membranes occurs at very early stages while NRF2 is activated later. Phosphorylation of p62 in its Ser351 residue occurs at early stages of phagocytosis and increases with time. We propose that once the RBC starts to degrade in the phagolysosome, and their contents are imported into the phagocyte cytosol for storage or recycling, phosphorylation of the p62 residue Ser351 increases, through an unknown mechanism, culminating with NRF2 translocation to the nucleus and induction of some of its transcriptional targets (Fig. 8F). In erythrophagocytosis, we could detect the up-regulation of three well-known NRF2-target genes: *p62*, *Ho-1* and *Sod2*. The results obtained for p62 confirm the positive feedback that exists between NRF2 and p62 that has been already reported for other experimental settings^43–45^. The up-regulation of the other two genes could result from the products of RBC degradation and reactive oxygen species formation. In erythrophagocytic macrophages, RBC processing is followed by heme release from hemoglobin and its subsequent translocation from the phagolysosome lumen into the cytoplasm, via a mechanism assisted by the heme responsive gene 1 transporter (HRG1)^16, 50, 51^. Once in the cytosol, the heme catabolism enzyme HO-1 releases iron from the protoporphyrin ring for storage or reuse. Thus, the increase in concentration of cytosolic heme may explain the increase of HO-1 expression. Besides iron extraction from protoporphyrin, HO-1 generates equimolar amounts of biliverdin and carbon monoxide (CO), two anti-oxidant molecules^52, 53^. *Sod2* up-regulation could be explained by an attempt of the macrophages to scavenge mitochondrial superoxide and thereby lower oxidative stress. Overproduction of reactive oxygen species has been described to be linked to impaired lysosomal function resulting from changes in cysteine residue of the Atp6v1a1 subunit of vATPase and its subsequent failure in acidifying the lysosomes^54–56^. Since lysosomal pH is a key determinant of lysosomal enzyme activity this could explain why in the absence of NRF2 or p62, RBC are not degraded. Finally, some autophagy genes (such as *Ndp52* and *Lc3b*) as well as *Lamp* were demonstrated to be upregulated by the NRF2 signaling pathway^57^ which in turn can contribute to the rapid degradation of RBC reinforcing the critical role of NRF2 in this process.

Although the mechanism via which the p62/NRF2 pathway modulates the degradation of RBC is far from being elucidated, with this work we reinforce the view that this non-canonical signaling pathway is activated in the absence of oxidative stress or under autophagic conditions^43–45, 58, 59^. Furthermore, we were able to identify new molecular machinery involved in erythrophagocytosis of RBC, opening new avenues for specific targeting and modulation of this process. This new knowledge may have a critical role in a number of hemolytic disorders associated with defects in RBC function that can lead to premature RBC senescence, such as sickle cell disease.

## Methods

### Cell culture

Rabbit vascular smooth muscle cells, used as a non-professional phagocytic cell line, were from ATCC (Manassas, VA, USA) and maintained in RPMI-1640 medium (Invitrogen, Carlsbad, CA, USA) containing 10% FBS and 100 U/mL antibiotics. Cells were grown in a humidified incubator at 37 °C under 5% CO_2_ atmosphere. Cells stably expressing Fcγ-RIIA were generated as described before^17, 60^. Cells were plated in 24-multiwell plates at a density of 30 × 10^3^ cells per well and grown on glass cover slips for 24 h. For experiments with the E1 inhibitor PYR-41 (Calbiochem, San Diego, CA, USA), 20 × 10^3^ cells were plated per well and 24 h after, the drug was added.

L929 cell line (kindly provided by Prof. Ira Tabas, Columbia University, NY, USA) was cultured to produce L-cell conditioned media (LCCM) enriched in M-CSF to differentiate monocytes into macrophages, as previously described^61^.

Bone marrow-derived macrophages (BMDM) were obtained from 8–12 week old C57BL/6J wild-type (WT), p62-knockout (kindly provided by Prof. Herbert Virgin, Washington University School of Medicine, St. Louis, MO, USA) and NRF2-KO mice. Primary macrophages were maintained as described^62^, but in RPMI-1640 medium, containing 10% HI-FBS and 30% LCCM. In the phospho-p62 experiments, BMDM were treated with 10 µM sodium arsenite (Sigma-Aldrich, St.Louis, MO, USA) for 12 h as positive control.

Mice were bred and maintained under specific pathogen-free (SPF) conditions, according to protocols approved by local (Instituto Gulbenkian de Ciência) and national (Portuguese Official Veterinary Department; Direcção Geral de Veterinária) ethics committees according to the Portuguese (Decreto-Lei 113/2013) and European (Directive 2010/63/EU) legislations. C57BL/6 *Nrf2* ^−/−^ mice were provided by the RIKEN BioResource Center (Koyadai, Tsukuba, Ibaraki, Japan). C57BL/6J wild-type and *Nrf2* ^−/−^ mice were co-housed from weaning (3–4 weeks old) to the date of the experiments.

Red blood cells (RBC) were obtained from human blood collected from healthy volunteers at CNC and CEDOC in accordance with protocols approved by ethics committees (Ethical Review Board of the Faculty of Medicine of the University of Coimbra and NOVA Medical School) and followed the Declaration of Helsinki. Written informed consent was obtained from all volunteers. Thus, all the protocols followed the portuguese and international guidelines. RBC were isolated and aged as described before^17^.

### Phagocytosis and phagosomal maturation assays

Aged RBC and IgG-opsonized latex beads were prepared as described before^17^ as well as phagocytosis and phagosomal maturation assessment (pulse-chase) experiments. In pulse-chase experiments, the pulse time was 30 min for non-professional phagocytes and 15 min for BMDM, followed by the chase times indicated in the graphs abscissa. Phagocytosis experiments with PYR-41 were performed as follows: the inhibitor was added to phagocytes at final concentrations of 5 and 10 μM, for experiments with RBC and IgG-opsonized particles, respectively, overnight. PYR-41 was present throughout the pulse-chase experiments. When the purpose of the experiment was the visualization of phagocytic cups, the phagocytic cells were challenged with phagocytic particles without synchronization and without RBC lysis.

### RNAi experiments

To knockdown p62 also known as SQSTM1 or Sequestosome and Rubicon in BMDM, a siRNA smart pool against p62, Rubicon and a non-targeting sequence siRNA, scramble (control) were used (GE Dharmacon, Lafayette, CO, USA). BMDM were transfected with Lipofectamine RNAiMAX (Invitrogen, Carlsbad, CA, USA) according to the manufacturer’s instructions. Experiments to assess RBC degradation were performed 72 h after transfection with siRNAs.

### Immunofluorescence and microscopy

Monoclonal antibodies used were: Lysosomal associated membrane protein-1 (LAMP-1) (1:50, Hybridoma Bank, Iowa City, IA, USA), Neighbor of BRCA1 gene1 [NBR1, (1:80, Abnova, Heidelberg, Germany)], Nuclear factor (erythroid-derived 2)-like 2 [NRF2, (1:50, Cell Signaling, Danvers, MA, USA)] and phospho(S351)-p62 (1:50, MBL Corporation, Japan). Polyclonal antibodies used were: LC3B (1:100, Cell Signaling, Taipei, Neihu, Taiwan), p62 C-term (1:80, Abgent, San Diego, CA, USA), Nuclear dot protein 52 [NDP52, (1:80, Abcam, Cambridge, UK)], and Ubiquitin (1:50, DAKO, Via Real Carpinteria, CA, USA). For immunoflourescence (IF), cells were fixed with 4% PFA for 30 min, permeabilized using 0.1% Triton X-100 (with 200 nM glycine) for 30 min and blocked with 0.5% Gelatin from cold water fish skin in PBS for 30 min. The exceptions were: LAMP-1 staining in which cells were permeabilized using methanol for 10 min; phospho-p62 in which cells were permeabilized for 10 min and no blocking was performed; NRF2 in which permeabilization and blocking were performed with 0.25% Triton X-100 and 1% BSA/10% FBS in 1X PBS/0.1% Tween-20, respectively. Then, the cells were incubated with the appropriate primary antibody for 90 min at room temperature (RT), followed by incubation with secondary antibody (1:800, from Jackson Immunoresearch, West Grove, PA, USA) for 1 h at RT. For visualization of phagocytic cups, phalloidin conjugated with Cy5 (1:100, Invitrogen), to stain F-actin, was added with the secondary antibodies. Stained samples were mounted with Mowiol/DABCO (Calbiochem, San Diego, CA, USA) and analyzed under a laser scanning confocal microscope (Carl Zeiss, Jena, Germany, LSM 510 software) or a Zeiss Cell Observer with a 63 × oil immersion objective (NA = 1.30). The images were analyzed by using LSM Image Browser, Image-J software or Zen software.

For live-cell imaging, BMDM were seeded in 35 mm glass bottom microwell petri dishes (MatTEK Corporation, Ashland, MA, USA) after differentiation and p62 silencing, BMDM were incubated for 15 min with RBC. After this time, non-internalized cells were lysed and the disappearance of Carboxyfluorescein-diacetate-Succinimidyl Ester (CFSE) fluorescence intensity was followed as a function of time under a Carl Zeiss LSM 710 META laser scanning confocal microscope (ZEN software) using a 63 × oil immersion objective (NA = 1.30), at 37 °C in CO_2_-independent medium. Fluorescence intensity of RBC containing phagosomes at 15 min pulse was normalized to 100.

### Quantitative PCR

*p62*, *Sod2*, *Ho-1* and *Rubicon* were assessed by quantitative real-time PCR (qPCR). Total RNA was isolated using the NZY Total RNA Isolation kit (NZYTech, Lisbon, Portugal), and 300 ng were reverse transcribed with iScript® cDNA synthesis kit (Bio-Rad, Hercules, CA, USA), according to the manufacturer’s protocols.

Primers sequence: *p62*, Forward 5′-GTCTTCTGTGCCTGTGCTGGAA-3′, Reverse 5′-TCTGCTCCACCAGAAGATCCCA-3′; *Ho-1*, Forward 5′-AAGGAGGTACACATCCAAGCCGAG-3′, Reverse 5′-GATATGGTACAAGGAAGCCATCACCAG-3′; *Sod2*, Forward 5′-TAAGGGTGGTGGAGAACCCAAAGGAG-3′, Reverse 5′-TTATTGAAGCCAAGCCAGCCCCAG-3′; *Rubicon*, Forward 5′-GAGGCCCCAGGAATATCACC-3′, Reverse 5′-GTGGGCGTTTTCCTTTTCCAG-3′; *Pgk1*, Forward 5′-ATGGATGAGGTGGTGAAAGC-3′, Reverse 5′-CAGTGCTCACATGGCTGACT-3′. Hypoxanthine phosphoribosyltransferase 1, *Hprt1*, (QIAGEN, Hilden, Germany) and *Pgk1* (Sigma-Aldrich, St.Louis, MO, USA) were used as housekeeping genes. The *p62, Sod2* and *Ho-1* mRNA levels were calculated by the Pfaffl method and normalized to both *Hprt1* and *Pgk1* mRNA levels.

### Western blot

For preparation of the total protein cell lysates, cells were lysed and blotted as described^63^. The antibodies incubated in TBS-Tween were: mouse p62 primary antibody (Clone 2C11, Abnova, Heidelberg, Germany) and ECL mouse HRP-conjugated secondary antibody (GE Healthcare, Little Chalfont, UK); mouse α-Tubulin primary antibody (Sigma-Aldrich) and Goat Anti-mouse HRP-conjugated secondary antibody (Bio-Rad, Hercules, CA, USA); and goat GAPDH primary antibody (Sicgene, Cantanhede, Portugal) and Rabbit Anti-Goat HRP-conjugated secondary antibody (Bio-Rad). For phospho-p62 blots, the protocol was based on the datasheet information. Mouse phospho-p62 primary antibody (M217-3, MBL Corporation, Japan) and ECL mouse HRP-conjugated secondary antibody (GE Healthcare, Little Chalfont, UK) were incubated in PBS-Tween (0.05%). Blots were developed with ECL (GE Healthcare). ChemiDoc^TM^ Touch Imaging System was used to detect fluorescence and bands quantification was performed using Image Lab software (Bio-Rad).

### Statistical analysis

Data are representative of at least three independent experiments. Unless stated otherwise, values depicted on graphs are expressed as mean ± SEM. Statistical analysis (t-test or Two-way ANOVA followed by Bonferroni post-test) was performed using the GraphPad PRISM software version. 5.0. *p* < 0.05 (*), *p* < 0.01 (**) and *p* < 0.001 (***) were considered to be statistically significant.

## Electronic supplementary material

Supplementary Information
