# Supplementary material for: Involvement of the p62/NRF2 signal transduction pathway on erythrophagocytosis
Source: Sci Rep. 2017 Jul 19;7:5812. doi: 10.1038/s41598-017-05687-1 (PMC5517431; doi:10.1038/s41598-017-05687-1)
Supplement: Supplementary file 1 — Supplementary Information [file 41598_2017_5687_MOESM1_ESM.pdf]

Involvement of the p62/NRF2 signal transduction pathway on erythrophagocytosis

Inês B. Santarino[1] (ines.santarino@nms.unl.pt), Michelle S. Viegas[2] (xelyviegas@yahoo.com.br), Neuza S. Domingues[1] (neuza.domingues@nms.unl.pt), Ana M. Ribeiro[3] (amribeiro@igc.gulbenkian.pt), Miguel P. Soares[3] (mpsoares@igc.gulbenkian.pt) and Otília V. Vieira[1,*] (otilia.vieira@nms.unl.pt)

SUPPLEMENTARY MATERIAL

SUP FIGURE 1: *Association of LC3B with phagosomal membranes in the absence of serum.*

Quantification of LC3B-II-positive phagosomes in non-professional phagocytes incubated in medium supplemented (black bars) or not/starved (grey bars) with serum, exposed to RBC and chased for the indicated times. The values are means ± SEM of, at least, three independent experiments. At each time point, at least, 100 phagosomes were analyzed.

SUP FIGURE 2: *Effect of Rubicon silencing on LCB-II phagosomal acquisition and assessment of p62 silencing in BMDM.*

(A) *Rubicon* mRNA fold change in knockdown versus control BMDM (transfected with scramble RNAi) was determined by RT-qPCR. Data were normalized to the endogenous *Hprt* and *Pgk1* genes. The values are means ± SEM expression levels of three independent experiments, each measured in two technical replicates. *, $p<0.05$ comparing differences between scramble and knockdown cells. (B) Quantification of LC3B-II-positive phagosomes in *Rubicon*-silenced- and in control-BMDM. Cells were exposed to RBC for 15 min and then chased for the indicated times. The values are means ± SEM of two independent experiments. ***, $p<0.001$; **, $p<0.01$.

(C) *p62* mRNA fold change in knockdown versus control BMDM (transfected with scramble RNAi) was determined by RT-qPCR. Data were normalized to the endogenous *Hprt* and *Pgk1* genes. The values are means ± SEM expression levels of four independent experiments, each measured in two technical replicates. ***, $p<0.001$ comparing differences between scramble and knockdown cells. (D) Western Blot of p62 expression levels in knockdown versus control cells (transfected with scramble RNAi). GAPDH was used as loading control.

SUP FIGURE 1

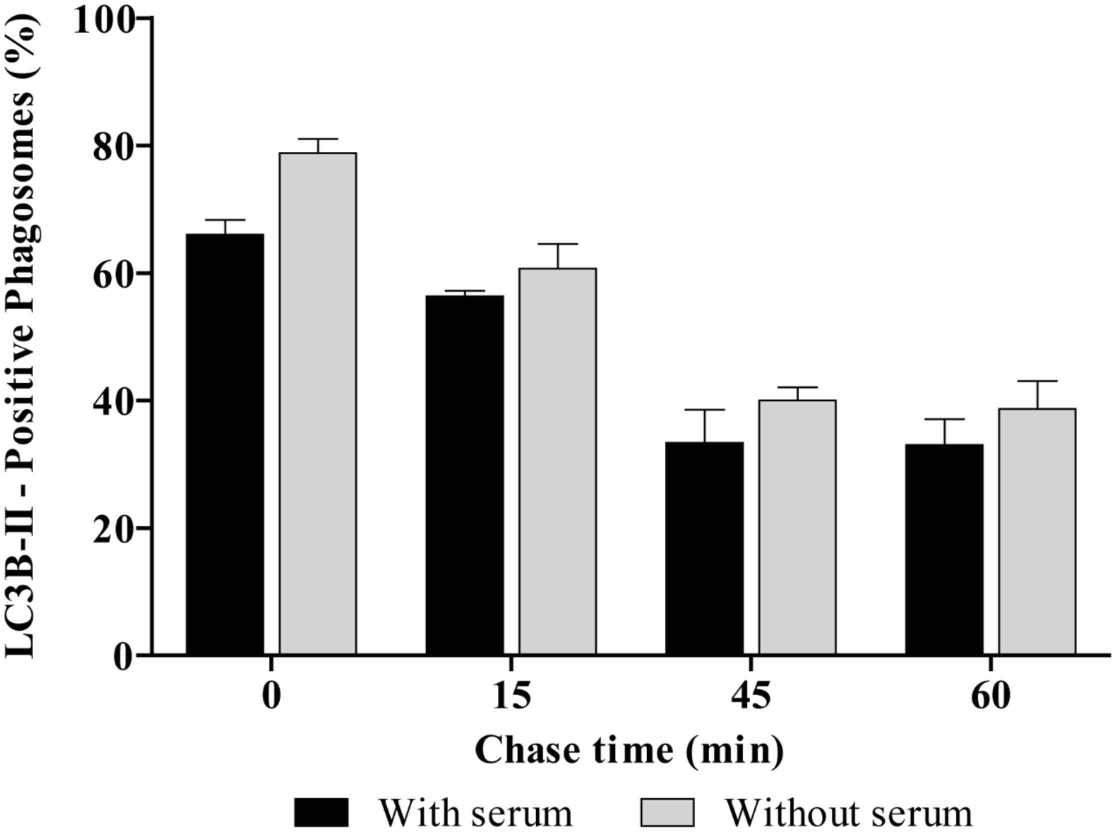

LC3B-II - Positive Phagosomes (%)
100
80
60
40
20
0
0
15
45
60
Chase time (min)
With serum
Without serum

SUP FIGURE 2

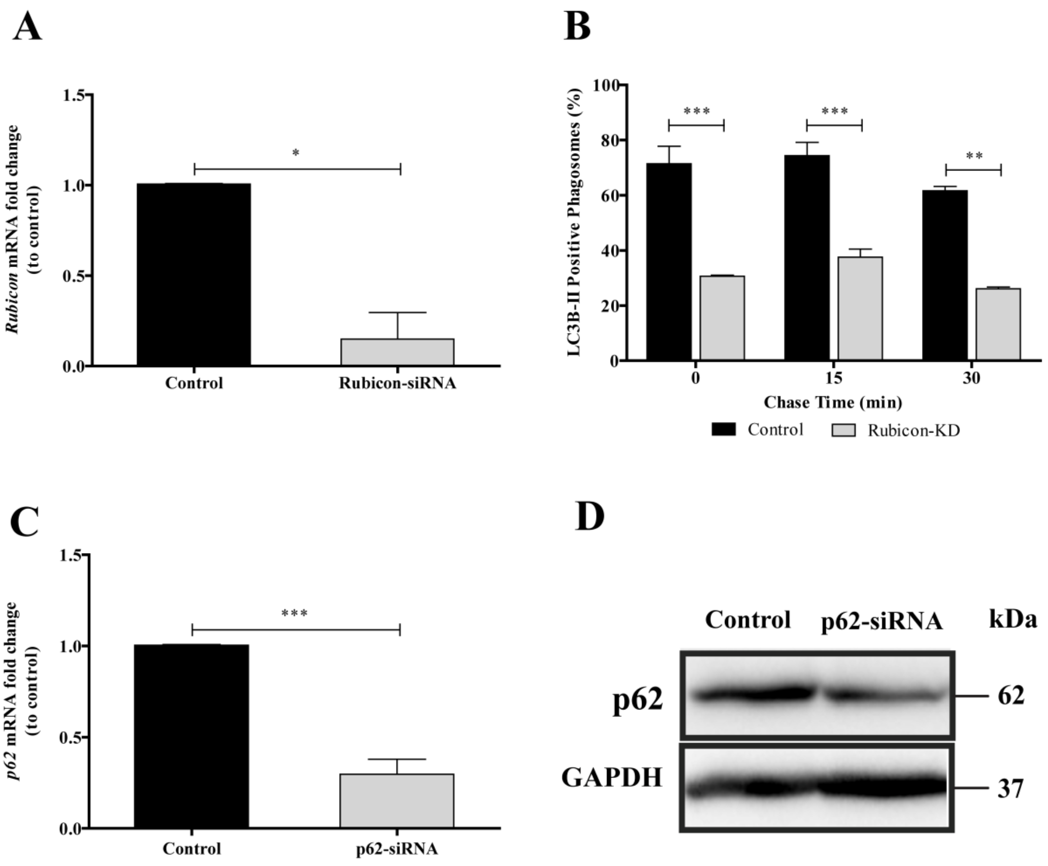
A
Rubicon mRNA fold change (to control)
1.5
1.0
0.5
0.0
*
Control
Rubicon-siRNA
B
LC3B-II Positive Phagosomes (%)
100
80
60
40
20
0
***
***
**
0
15
30
Chase Time (min)
Control
Rubicon-KD
C
p62 mRNA fold change (to control)
1.5
1.0
0.5
0.0
***
Control
p62-siRNA
D
Control
p62-siRNA
kDa
p62
62
GAPDH
37
